# The Foot Fault Scoring System to Assess Skilled Walking in Rodents: A Reliability Study

**DOI:** 10.3389/fnbeh.2022.892010

**Published:** 2022-04-29

**Authors:** Lucas Athaydes Martins, Aniuska Schiavo, Léder Leal Xavier, Régis Gemerasca Mestriner

**Affiliations:** ^1^Graduate Program in Biomedical Gerontology, School of Medicine, Pontifical Catholic University of Rio Grande do Sul (PUCRS), Porto Alegre, Brazil; ^2^Neurorehabilitation and Neural Repair Research Group (NEUROPLAR), Pontifical Catholic University of Rio Grande do Sul, Porto Alegre, Brazil; ^3^School of Health and Life Sciences, Pontifical Catholic University of Rio Grande do Sul, Porto Alegre, Brazil

**Keywords:** locomotion, rodentia, reliability, rat, mice, walking

## Abstract

The foot fault scoring system of the ladder rung walking test (LRWT) is used to assess skilled walking in rodents. However, the reliability of the LRWT foot fault score has not been properly addressed. This study was designed to address this issue. Two independent and blinded raters analyzed 20 rats and 20 mice videos. Each video was analyzed twice by the same rater (80 analyses per rater). The intraclass correlation coefficient (ICC) and the Kappa coefficient were employed to check the accuracy of agreement and reliability in the intra- and inter-rater analyses of the LRWT outcomes. Excellent intra- and inter-rater agreements were found for the forelimb, hindlimb, and both limbs combined in rats and mice. The agreement level was also excellent for total crossing time, total time stopped, and the number of stops during the walking path. Rating individual scores in the foot fault score system (0–6) ranged from satisfactory to excellent, in terms of the intraclass correlation indexes. Moreover, we showed that experienced and inexperienced raters can obtain reliable results if supervised training is provided. We concluded that the LRWT is a reliable and useful tool to study skilled walking in rodents and can help researchers address walking-related neurobiological questions.

## Introduction

Skilled walking is crucial for walking adaptability, i.e., a complex sensory-motor function, qualified or required to control and coordinate various degrees of freedom in joints, in a variety of environmental contexts, or that interfere with locomotion (Houdijk et al., 2012; Balasubramanian et al., 2014; Ducharme et al., 2018). Gait is influenced by the temporal and spatial integration of the cognitive and neuromusculoskeletal neural systems (Shimada et al., 2013). Moreover, the ability to adapt gait according to environmental context is a crucial aspect in maintaining body stability and preventing falls (Van Ooijen et al., 2015; Esteves et al., 2016; Caetano et al., 2018; Medeiros et al., 2018). The ability to adapt walking demands skilled movements during locomotion, which is frequently impaired in the injured nervous system. While automatic walking is mainly brain stem and spinal cord-dependent, the skilled movements needed to adapt walking in challenging pathways require higher cerebral cortex processing (Farr et al., 2006; Ueda et al., 2018). Thus, central nervous system diseases or injuries can limit the ability to adapt walking (Mestriner et al., 2013).

Although several studies into skilled walking have focused on human biomechanics (Hawkins et al., 2017; Lanini et al., 2017; Ducharme et al., 2018), animal models can usefully provide neurobiological insights at the cellular and molecular levels (Soleman et al., 2010; Mestriner et al., 2013; Wearick-Silva et al., 2018). For instance, the ladder rung walking test (LRWT) has been used to assess skilled walking (Metz and Whishaw, 2002, 2009) in unilateral ischemic injury in the motor cortex (Soleman et al., 2010; Antonow-Schlorke et al., 2013); spinal cord injury (Sandner et al., 2018; Guo et al., 2019); dopaminergic depletion induced by 6-hydroxydopamine (a model of Parkinson’s disease) (Faraji and Metz, 2007); neonatal white matter injury (Ueda et al., 2018); and stress-related conditions (Metz et al., 2015; Medeiros et al., 2018; Wearick-Silva et al., 2018).

In the LRWT, walking adaptability is assessed using a foot fault score that reflects rodent walking pattern, inter-foot coordination, foot support, forelimb and hindlimb kinematics, step and gait cycles, and gait speed (Antonow-Schlorke et al., 2013; Guo et al., 2019). The test apparatus consists of plexiglass walls and metal rungs that are inserted to create a floor, thus forming a horizontal ladder. Rodents are expected to cross the ladder using the available rungs to support paw placement. The better the ability to adapt walking, the lower the number of faults (slips and falls) while crossing the apparatus (Metz and Whishaw, 2002, 2009). The foot fault score system is a 7-point category scale in which the quality and appropriateness of foot placement are judged by analyzing a video recording, frame by frame, of rodents walking along a 1-m-long horizontal ladder. The rungs are arranged in a pattern that requires murine ability to adapt walking (Metz and Whishaw, 2002, 2009). This scoring system requires only a hand camera and a minimally trained researcher to analyze the video and apply the foot fault score (Metz and Whishaw, 2002; Wearick-Silva et al., 2018). This method may avoid common pitfalls that occur when using reflective markers on the flexible skin of rodents (Filipe et al., 2006; Bauman and Chang, 2010) and gives a measure of the success in adapting walking (Medeiros et al., 2018; Wearick-Silva et al., 2018).

However, to the best of our knowledge, the foot fault score has not been properly assessed regarding its intra- and inter-rater reliability and reproducibility, which is a source of uncertainty. Current studies usually elect a single rater to analyze all videos in an attempt to minimize bias, which is scientifically insufficient. We hypothesized that it is sufficient for raters to read and understand the instructions provided by the foot fault score creators to ensure reliable measures (Metz and Whishaw, 2002, 2009). Thus, this study sought to compare the scores obtained by two raters, one experienced and another without previous experience in applying the foot fault score. This article provides scientific information regarding the external validity of the LRWT findings in rodents, thus contributing to advance the field of skilled walking neurobiology.

## Materials and Methods

We randomly selected 40 video recordings of rodents from our laboratory database (20 recordings of Wistar CrlCembe: WI rats and 20 recordings of C57BL/6JUnib mice), which performed the horizontal LRWT. At the time of the original experiments, the animals were provided by the Center for Experimental Biological Models (CeMBE) of the Pontifical Catholic University of Rio Grande do Sul. The animals were housed in cages, each containing three to four rodents on a 12-h dark–light cycle with food and water available *ad libitum*, at a temperature of 22–24°C. The experiments were carried out in accordance with the National Council for Animal Control and Experimentation (Concea), and all the procedures were approved by the University Animal Ethics Commission (CEUA) under protocol numbers 15/00442 and 15/00475.

### Ladder Rung Walking Test

**FIGURE 1:**
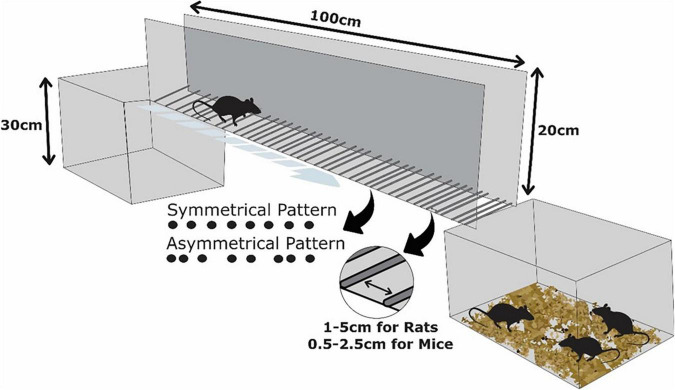
Schematic illustration of the ladder rung walking test apparatus. Note that the rungs could be inserted in symmetrical or asymmetrical patterns. The between-rung space could be varied from 1 to 5 cm for rats and from 0.5 to 2.5 cm for mice. The animal starts the trial from the empty box and is expected to walk to achieve the home cage.

We used two LRWT apparatuses, one for rats and another one adapted for mice. Both consisted of clear plexiglass side walls (100 cm long and 20 cm high). The diameter of the metal rungs varied, being 3 mm for rats and 2 mm for mice. The minimum and maximum gaps between the rungs also varied, being from 1 to 5 cm for rats and from 0.5 to 2.5 cm for mice. In both cases, the ladders were elevated horizontally 30 cm above the ground, with a neutral cage placed in the starting position and the animal’s home cage placed at the opposite end of the ladder (Figure 1). The between-wall distance was adjusted leaving 1 cm wider than the size of the rodent to prevent the animal from turning around during the crossing (Metz and Whishaw, 2002; Farr et al., 2006; Wearick-Silva et al., 2018).

The pattern of the metal rungs demands different degrees of skilled walking and can be used to vary the complexity of the test. A regular arrangement allows animals to learn the position of the rungs over training sessions and to anticipate limb placement (Figure 1, symmetrical pattern). In irregular patterns, rungs are randomly repositioned in each trial to prevent the rodents from learning the rung sequence. Thus, irregular patterns are more useful when studying skilled walking (Figure 1, asymmetrical pattern) (Metz and Whishaw, 2002; Medeiros et al., 2018; Wearick-Silva et al., 2018). In this study, only irregular rung patterns were analyzed.

In the test, the animals were placed at the beginning of the ladder, and walked along with it, adapting their foot placement on the rungs until reaching the home cage (Figure 1). While performing the test, we filmed the rodents using a camera (GoPro Hero 4, 12 megapixels). An acquisition rate of 240 frames per second (FPS) in a lateral view was adopted, allowing a *post hoc* frame-by-frame video analysis.

### Foot Fault Scoring System Ladder Rung Walking Test

To assess the forelimb and hindlimb placement on the rungs, which requires precise and coordinated foot positioning as well as stride and inter-limbic coordination, a quantitative foot fault scoring system (Metz and Whishaw, 2002) derived from a categorical analysis was used. In the system, a frame-by-frame video recording analysis is performed to identify the steps in each limb and qualify foot placement using a 7-point category scale (Metz and Whishaw, 2002, 2009; Table 1). The score 0 is given when the limb did not touch the rung (missed a rung) and resulted in a fall (total miss). A fall is considered when the limbs fell between rungs and the animal’s posture and balance are disturbed. Score 1 is given when the limb slipped off a rung and a fall occurred (deep slip). Score 2 is given when the limb slipped off a rung during weight bearing, but a fall did not occur and the rodent interrupts walking (slight slip). Score 3 is given when, before weight bearing the limb on a rung, the rodent quickly lifted and placed the foot on another rung (replacement). Score 4 occurs when the limb is clearly about to be placed on a rung, but the rodent quickly changes the feet placement to another rung without touching the first rung (correction). Score 4 is also given when the limb is placed on a rung, but the animal removes the foot and repositions it on the same rung. Score 5 is given when the limb is placed on the rung either using the wrist or digits for the forelimb or heel or toes for the hindlimb (partial placement). Finally, score 6 is given when the full body weight bearing is applied on a rung with the midportion of the foot (correct placement) (Table 1).

**TABLE 1:** Rating scale for foot placement in the LRWT.

Category	Type of foot misplacement
0	Total miss
1	Deep slip
2	Slight slip
3	Replacement
4	Correction
5	Partial placement
6	Correct placement

The score given in each category is then multiplied by the frequency of foot placements in the same category. Later, the sum of all the categories provides the total combined score (sum of the forelimb plus the hindlimb scores). The fully explained video protocol and all technical details to apply the foot fault score were previously published by Metz and Whishaw (2009).

In this study, the following outcomes in the LRWT were assessed for inter- and intra-rater agreements: total crossing time, number of stops, total time stopped, scores 0–6 for forelimb, total score for forelimb, scores 0–6 for hindlimb, total score of the hindlimb, and the combined total score of limbs.

The skilled walking performance score (SWPS) was represented as a percentage of the maximum possible performance (100%) (Medeiros et al., 2018; Altamentova et al., 2020). The number of cycles (NC) each rodent took to cross the ladder was multiplied by 6 (the maximum score for each cycle in the foot fault score system) and the resulting number was considered the maximum possible performance of each animal in a trial (100%). Then, during a trial, each cycle was rated according to the foot fault score system and the sum of the obtained scores provided the total score in the trial (TS). Finally, the SWPS was represented as a percentage of the maximum possible performance (100%) (Medeiros et al., 2018; Altamentova et al., 2020), as follows:


$$S\,W\,P\,S=\frac{T\,S\times 100}{N\,C\times 6}$$

where *SWPS* is skilled walking performance score, *TS* is total score in the trial, *NC* is the number of cycles, and 6 is the maximum score for each cycle in the foot fault score system.

### Foot Placement Reliability Between Inter- and Intra-Rater

To assess inter- and intra-rater reliability, two independent and blinded raters (called I and II) analyzed 20 rats and 20 mice videos. Each video was analyzed twice by the same rater (80 analyses per rater). The videos were named randomly by another independent researcher (not involved in the analyses) to prevent raters I and II from perceiving half of the videos that were similar. Thus, each video had a different number to ensure a blinded reproducibility Analysis. Rater I (A. Schiavo) was inexperienced in the foot fault score and received supervised training before starting data collection. Rater II (L. A. Martins) had previous experience and publications using the LRWT (Medeiros et al., 2018; Wearick-Silva et al., 2018).

### Statistical Analysis

Descriptive statistics were used to characterize the sample profile in the SWPS. The intraclass correlation coefficient (ICC) and the Kappa coefficient were employed to verify the accuracy of agreement and reliability in the inter- and intra-rater analyses of the foot fault scores. Agreement values in ICC greater than 0.75 were considered “excellent,” those between 0.4 and 0.75 were considered “satisfactory,” and those <0.4 were considered “poor.” When negative ICC values (difference between values greater than sample variance) occurred, the data were replaced by zero, as recommended by Bartko (1976) and Shrout and Fleiss (1979). The statistical analysis was performed using the software Statistical Package for the Social Sciences (SPSS) version 20.0.

## Results

### Inter-Rater Reliability for Rat

The LRWT analyses in rats demonstrated that raters I and II achieved an excellent agreement in the combined total score of limbs (ICC = 0.938/*p* = 0.0001). Regarding all the timed outcomes, the total crossing time (ICC = 0.994/*p* = 0.0001) and the total time stopped (ICC = 0.992/*p* = 0.0001) agreement levels were considered excellent, as were the variable number of stops (ICC = 0.957/*p* = 0.0001). Thus, the reliability between the total score for forelimb and hindlimb placement was shown to be excellent.

Furthermore, we analyzed the reliability among all scores described in the test, specifically, in the categories of 0–6 for each of the limbs evaluated. For the forelimb, the data showed an excellent reliability for scores 0, 1, and 2, with ICC varying from 0.839 to 1 (*p* = 0.0001) as well as for scores 5 and 6, with ICC varying from 0.813 and 0.854, respectively (*p* = 0.0001). However, for the forelimb scores 3 and 4, the raters obtained a satisfactory agreement (ICC 0.721 and 0.551, respectively/*p* ≤ 0.045). Similarly, for the hindlimb, excellent reliability was obtained for scores 0, 1, 3, and 4, with ICC ranging from 0.889 to 0.931 (*p* = 0.0001). The reliability for scores 2, 5, and 6 was also considered satisfactory (Table 2).

**TABLE 2:** Agreement between raters I and II regarding the outcomes obtained in the LRWT in rats.

Outcome	ICC (IC 95%)	Cronbach’s alpha	*p*-Value
**Foot fault scoring in rat**			
Combined total score	0.938 (0.844–0.976)	0.938	0.0001*
Total crossing time	0.994 (0.985–0.998)	0.994	0.0001*
Number of stops	0.957 (0.892–0.983)	0.957	0.0001*
Total time stopped	0.992 (0.980–0.997)	0.992	0.0001*
**Forelimb placement**			
Score 0	1 (1–1)	1	0.0001*
Score 1	0.839 (0.594–0.936)	0.839	0.0001*
Score 2	0.903 (0.754–0.961)	0.903	0.0001*
Score 3	0.721 (0.295–0.889)	0.721	0.004*
Score 4	0.551 (0.000–0.822)	0.551	0.045*
Score 5	0.854 (0.631–0.942)	0.854	0.0001*
Score 6	0.813 (0.528–0.926)	0.813	0.0001*
Total score	0.879 (0.695–0.952)	0.879	0.0001*
**Hindlimb placement**			
Score 0	0.889 (0.719–0.956)	0.889	0.0001*
Score 1	0.931 (0.826–0.973)	0.931	0.0001*
Score 2	0.593 (0.000–0.839)	0.593	0.028*
Score 3	0.889 (0.719–0.956)	0.889	0.0001*
Score 4	0.889 (0.719–0.956)	0.889	0.0001*
Score 5	0.41 (0.000–0.620)	0.41	0.456
Score 6	0.592 (0.000–0.839)	0.592	0.029*
Total score	0.931 (0.826–0.973)	0.931	0.0001*

*Statistically significant difference.

**FIGURE 2:**
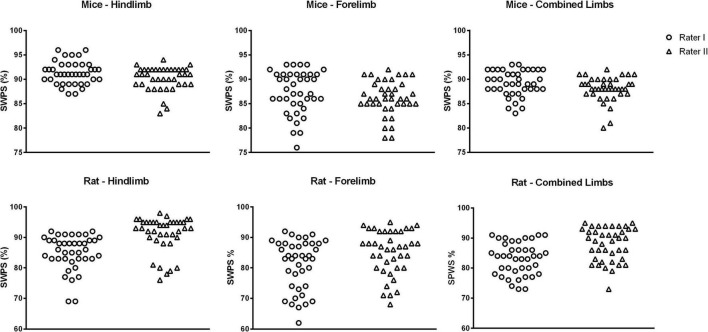
Skilled walking performance score (SWPS)* obtained by raters I and II. The SWPS is represented as a percentage of the maximum possible performance. The number of cycles (NC) each rodent took to cross the ladder is multiplied by 6 (the maximum score for each cycle in the foot fault score system) and the resulting number is considered the maximum possible animal performance (100%). In a trial, each cycle is rated according to the foot fault score system and the sum of the obtained scores provided the total score in the trial (TS). *SWPS = [(TS × 100)/(NC × 6)].

**FIGURE 3:**
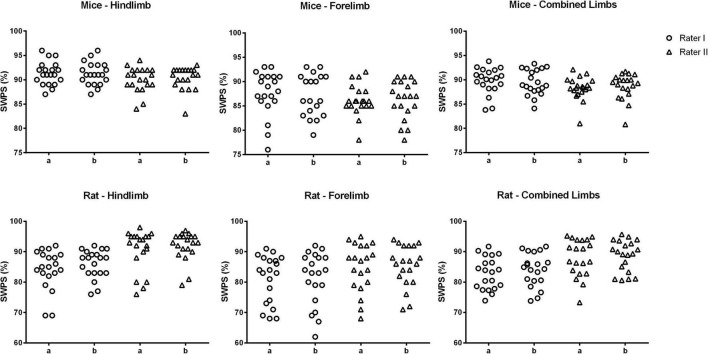
Skilled walking performance score (SWPS) obtained at first (a) and second (b) assessment by raters I and II. Note that both assessments present the same data distribution pattern.

**FIGURE 4:**
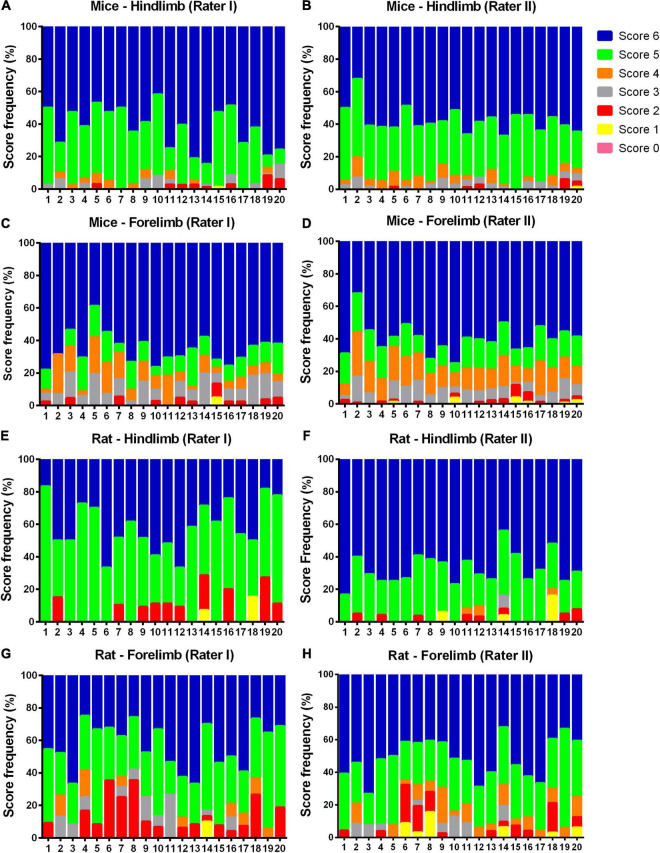
Frequency (%) of each foot placement rate (from 0 to 6), as judged by raters I and II in the study rodents. Note that all possible foot placement rates were observed in the study sample, while score 6 (correct placement) was the most frequent for both forelimb and hindlimb, and the lower scores (≤3) were also present. **(A–D)**: mice hindlimb and forelimb rates; **(E–H)**: rat hindlimb and forelimb rates.

**FIGURE 5:**
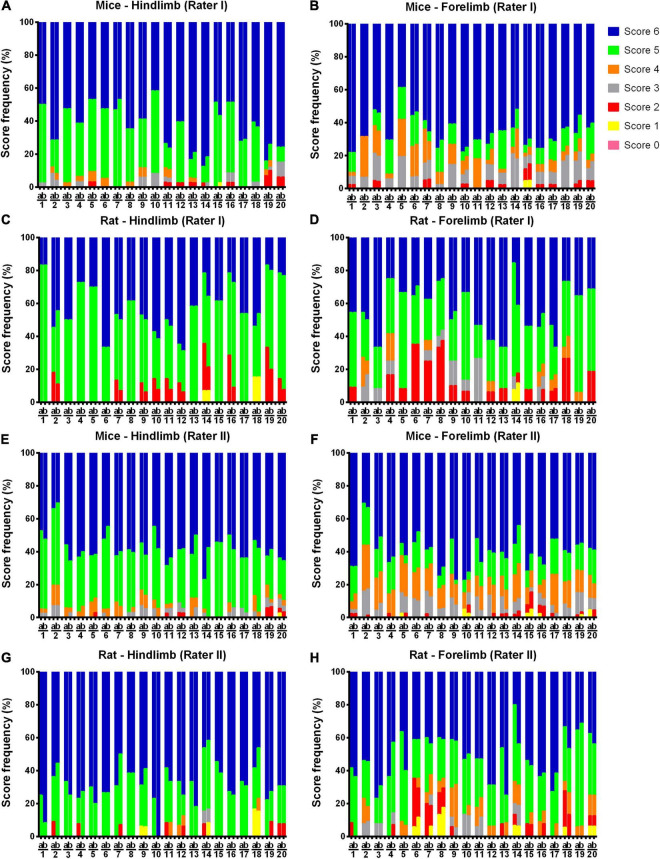
Frequency (%) of each foot placement rate (from 0 to 6) in the first (a) and second (b) assessment by raters I and II. Note that the overall rate distribution pattern was similar between raters and trials. **(A–D)**: scores attributed by rater I; **(E–H)**: scores attributed by rater II.

The individual results for each animal in relation to SWPS are shown in Figures 2, 3. In addition, the frequency of each score (1–6) for the hindlimb and forelimb of each rodent is shown in Figures 4, 5.

### Inter-Rater Reliability for Mice

The inter-rater reliability score system for mice is shown in Table 3. We observed a strong agreement between the raters in the combined total score (ICC = 0.954/*p* = 0.0001), total crossing time (ICC = 1/*p* = 0.0001), the number of steps (ICC = 0.922/*p* = 0.0001), and total time stopped (ICC = 0.998/*p* = 0.0001). In addition, the forelimb and hindlimb placement scores showed excellent agreement in the LRWT, with less consistency for forelimb placement (score 3) (ICC = 0.466/*p* = 0.090) and hindlimb correction (score 4) (ICC = 0.484/*p* = 0.079). Overall, the total scores for the forelimb (ICC = 0.925/*p* = 0.0001) and hindlimb (ICC = 0.919/*p* = 0.0001) placement between raters I and II showed strong agreement.

**TABLE 3:** Agreement between raters I and II regarding the outcomes (scores) recorded in the LRWT in mice.

Outcome	ICC (IC 95%)	Cronbach’s alpha	*p*-Value
**Foot fault scoring in mice**			
Combined total score	0.954 (0.883–0.982)	0.954	0.0001*
Total crossing time	1 (0.999–1)	1	0.0001*
Number of stops	0.922 (0.802–0.969)	0.922	0.0001*
Total time stopped	0.998 (0.995–0.999)	0.998	0.0001*
**Forelimb placement**			
Score 0	0.889 (0.719–0.956)	0.889	0.0001*
Score 1	0.755 (0.381–0.903)	0.755	0.002*
Score 2	0.699 (0.239–0.881)	0.699	0.006*
Score 3	0.466 (0.000–0.789)	0.466	0.090
Score 4	0.904 (0.757–0.962)	0.904	0.0001*
Score 5	0.830 (0.571–0.933)	0.830	0.0001*
Score 6	0.712 (0.271–0.886)	0.721	0.005*
Total score	0.925 (0.812–0.970)	0.925	0.0001*
**Hindlimb placement**			
Score 0	0.822 (0.550–0.929)	0.822	0.0001*
Score 1	0.889 (0.719–0.956)	0.889	0.0001*
Score 2	0.938 (0.844–0.976)	0.938	0.0001*
Score 3	0.751 (0.371–0.901)	0.751	0.002*
Score 4	0.484 (0.000–0.796)	0.484	0.079
Score 5	0.622 (0.046–0.850)	0.622	0.0001*
Score 6	0.764 (0.405–0.907)	0.764	0.001*
Total score	0.919 (0.795–0.968)	0.919	0.0001*

*Statistically significant difference.

### Intra-Rater Reliability for Rat

Table 4 shows the intra-rater analyses in rats. We found excellent agreement in the combined total score, total crossing time, number of stops, and total time stopped for both raters. Regarding score evaluation, rater I obtained excellent agreement in all the scores for the forelimb (ICC between 0.899 and 0.989/*p* = 0.0001). Rater II achieved excellent agreement in all scores for forelimb (ICC between 0.787 and 0.920), except for score 6, which was considered satisfactory (ICC = 0.652/*p* = 0.13).

**TABLE 4:** Intra-rater agreement on outcomes in the analyses of the LRWT in rats.

	Rater I			Rater II		
Outcome	ICC (IC 95%)	Cronbach’s alpha	*p*-Value	ICC (IC 95%)	Cronbach’s alpha	*p*-Value
Combined total score	0.969 (0.922–0.988)	0.969	0.0001*	0.950 (0.875–0.980)	0.950	0.0001*
Total crossing time	0.993 (0.982–0.997)	0.993	0.0001*	0.981 (0.953–0.993)	0.981	0.0001*
Number of stops	0.950 (0.873–0.980)	0.950	0.0001*	0.915 (0.786–0.966)	0.915	0.0001*
Total time stopped	0.806 (0.509–0.923)	0.806	0.0001*	0.939 (0.847–0.976)	0.939	0.0001*
**Forelimb placement**						
Score 0	0.899 (0.719–0.956)	0.899	0.0001*	0.919 (0.796–0.968)	0,919	0.0001*
Score 1	0.889 (0.719–0.956)	0.889	0.0001*	0.842 (0.600–0.937)	0.842	0.0001*
Score 2	0.989 (0.973–0.996)	0.989	0.0001*	0.877 (0.688–0.951)	0.877	0.0001*
Score 3	0.978 (0.944–0.991)	0.978	0.0001*	0.920 (0.797–0.968)	0.920	0.0001*
Score 4	0.941 (0.851–0.977)	0.941	0.0001*	0.849 (0.618–0.940)	0.849	0.0001*
Score 5	0.948 (0.869–0.979)	0.948	0.0001*	0.787 (0.462–0.916)	0.787	0.001*
Score 6	0.905 (0.761–0.963)	0.905	0.0001*	0.652 (0.121–0.862)	0.652	0.13
Total score	0.916 (0.787–0.967)	0.916	0.0001*	0.875 (0.685–0.951)	0.875	0.0001*
**Hindlimb placement**						
Score 0	1 (1–1)	1	0.0001*	1 (1–1)	1	0.0001*
Score 1	1 (1–1)	1	0.0001*	0.962 (0.904–0.985)	0.962	0.0001*
Score 2	0.838 (0.591–0.936)	0.838	0.0001*	0.829 (0.567–0.932)	0.829	0.0001*
Score 3	1 (1–1)	1	0.0001*	1 (1–1)	1	0.0001*
Score 4	1 (1–1)	1	0.0001*	0.904 (0.758–0.962)	0.904	0.0001*
Score 5	0.992 (0.980–0.997)	0.992	0.0001*	0.637 (0.83–0.856)	0.637	0.16
Score 6	0.982 (0.954–0.993)	0.982	0.0001*	0.810 (0.519–0.925	0.810	0.0001*
Total score	0.988 (0.970–0.995)	0.988	0.0001*	0.970 (0.924–0.988)	0.970	0.0001*

*Statistically significant difference.

In relation to hindlimb agreement, rater I obtained a similar excellent degree of agreement to that for the forelimb, with ICC ranging from 0.838 to 1/*p* = 0.0001. In contrast, rater II achieved a lower agreement than rater I, and the ICC was very good, ranging from 0.637 to 1, with only score 5 graded as satisfactory (ICC 0.637). Moreover, both raters obtained excellent intra-rater scores in the outcomes, namely, combined total score, total crossing time, number of steps, total time stopped, and total score for forelimb and hindlimb, with ICC ranging from 0.806 to 0.993 for rater I and from 0.915 to 0.981 for rater II.

### Intra-Rater Reliability for Rats

Overall, the intra-rater reliability for mice was excellent for both raters (Table 5). For rater I, in the forelimb, foot placement agreement for all the 7 scores was excellent (with ICC ranging from 0.939 to 1/*p* = 0.0001). For rater II, the agreement was also excellent, with ICC varying between 0.778 and 0.968 for scores 0–5. However, score 6 was considered satisfactory (ICC 0.488/*p* = 0.077). Regarding the hindlimb placement, similar results were found, with the raters only differing in score 6 (rater II obtained a lower ICC: 0.749/*p* = 0.002) (Table 5).

**TABLE 5:** Intra-rater agreement on outcomes in the analyses of the LRWT in mice.

	Rater I			Rater II		
Outcome	ICC (IC 95%)	Cronbach’s alpha	*p*-Value	ICC (IC 95%)	Cronbach’s alpha	*p*-Value
Combined total score	0.971 (0.926–0.988)	0.971	0.0001*	0.963 (0.906–0.985)	0.963	0.0001*
Total crossing time	1 (1–1)	1	0.0001*	0.999 (0.998–1)	0.999	0.0001*
Number of stops	0.948 (0.868–0.979)	0.948	0.0001*	0.774 (0.429–0.911)	0.774	0.001*
Total time stopped	0.988 (0.969–0.995)	0.988	0.0001*	0.985 (0.963–0.994)	0.985	0.0001*
**Forelimb placement**						
Score 0	1 (1–1)	1	0.0001*	0.919 (0.796–0.968)	0.919	0.0001*
Score 1	1 (1–1)	1	0.0001*	0.778 (0.440–0.912)	0.778	0.001*
Score 2	0.979 (0.947–0.992)	0.979	0.0001*	0.829 (0.568–0.932)	0.829	0.0001*
Score 3	0.979 (0.948–0.992)	0.979	0.0001*	0.899 (0.746–0.960)	0.899	0.0001*
Score 4	0.939 (0.846–0.976)	0.939	0.0001*	0.956 (0.888–0.982)	0.956	0.0001*
Score 5	0.982 (0.965–0.995)	0.982	0.0001*	0.928 (0.817–0.971)	0.928	0.0001*
Score 6	0.950 (0.873–0.980)	0.950	0.0001*	0.488 (0.000–0.797)	0.488	0.077
Total score	0.978 (0.944–0.991)	0.978	0.0001*	0.934 (0.833–0.974)	0.934	0.0001*
**Hindlimb placement**						
Score 0	0.919 (0.796–0.968)	0.919	0.0001*	1 (1–1)	1	0.0001*
Score 1	0.889 (0.719–0.956)	0.889	0.0001*	0.889 (0.719–0.956)	0.889	0.0001*
Score 2	0.978 (0.945–0.991)	0.978	0.0001*	0.963 (0.908–0.986)	0.936	0.0001*
Score 3	0.936 (0.839–0.975)	0.936	0.0001*	0.821 (0.548–0.929)	0.821	0.0001*
Score 4	1 (1–1)	1	0.0001*	0.886 (0.713–0.955)	0.886	0.0001*
Score 5	0.982 (0.953–0.993)	0.982	0.0001*	0.861 (0.649–0.945)	0.861	0.0001*
Score 6	0.958 (0.895–0.983)	0.958	0.0001*	0.749 (0.367–0.901)	0.749	0.002*
Total score	0.950 (0.873–0.980)	0.950	0.0001*	0.951 (0.876–0.981)	0.951	0.0001*

*Statistically significant difference.

## Discussion

Studying skilled walking in rodents is of importance to translational neuroscience, since the irregular distribution of the rungs in the walking path requires the animal’s capacity to adjust its stride length and paw placement and control the center of mass. These adaptive motor control strategies are also found and widely studied in humans (Beauchet et al., 2008). Rodents and humans perform some similar movements to protect an injured limb and/or prevent falls (Jacobs et al., 2014). The LRWT fulfills the fundamental principles of skilled walking, such as pattern of rhythmic reciprocal limb movement, supporting body balance against gravity and adapting locomotion in response to environmental challenges (Balasubramanian et al., 2014).

Metz and Whishaw created the LRWT in 2002 to assess forelimb and hindlimb stepping, placing, and coordination in models of cortical and subcortical injury. According to the authors, the test is a sensitive skilled task for assessing slight impairments of walking function and is useful when assessing functional recovery following brain or spinal cord injury and the effectiveness of rehabilitative therapies (Metz and Whishaw, 2002; Riek-Burchardt et al., 2004). Locomotion during the LRWT is known to depend on ascending and descending neural pathways, since accurately crossing the rungs requires finely adjusted motor control, balance, limb coordination, and muscle control (Metz and Whishaw, 2002; Medeiros et al., 2018; Wearick-Silva et al., 2018).

However, to determine the psychometric properties of behavioral tests, it is essential to obtain reliable, consistent, and scientifically valid findings (Souza et al., 2017). Both intra- and inter-rater agreements are important metrics to ensure reliability and reproducibility (Meseguer-Henarejos et al., 2018). Here, we sought to assess intra- and inter-rater agreement in the foot fault score of the LRWT using two strains of rodents, i.e., Wistar rats and C57BL/6 mice. Two independent researchers (with and without previous experience using the test’s scoring system) analyzed the videos. Our findings suggest that the foot fault score system of the LRWT is a useful, reliable, and consistent tool for studying skilled walking performance in rodents. We also found excellent inter and intra-rater reliability for “total crossing time,” “number of stops,” and “total time stopped.” The agreement measures provided by this study suggest that data obtained by different research groups using the LRWT should be comparable (Hutchinson et al., 2017) and should encourage the use of the test in further studies.

The LRWT is an interesting option for researchers investigating neural mechanisms involved in the ability to adapt walking (Metz et al., 2001; Faraji and Metz, 2007; Medeiros et al., 2018; Wearick-Silva et al., 2018). Since the score reflects the animal’s ability to adapt limb placement and position in a contextual environment (Metz and Whishaw, 2002; Jacobs et al., 2014), the foot fault score system is useful to study skilled walking in rodents (Medeiros et al., 2018; Wearick-Silva et al., 2018). While traditional biomechanical models of walking analysis require expensive devices, constant animal handling for placing reflective markers, and development of signal-processing routines (Vrinten and Hamers, 2003; Berryman et al., 2009), the LRWT provides skilled walking assessment using a fast, simple, and inexpensive method.

Whereas we observed satisfactory to excellent intraclass correlation indexes in rating individual scores (0–6), caution is necessary when using the foot fault score system. Individual scores present subtle differences that may confuse untrained raters. For example, differentiating between scores 3 (replacement) and 4 (correction) requires attention to identify whether the rodent touched the rung before completing paw placement. Moreover, in some situations, the rodent supports a single paw simultaneously on two rungs that are placed too close to each other. This may cause confusion in scores 5 (partial placement) or 6 (correct placement). In addition, rodents sometimes place their paw on the acrylic wall to help walking forward, a behavior that is not considered in the foot fault score system. Furthermore, the subtle differences between scores 1 (deep slip) and 2 (slight slip) may cause uncertainty for untrained raters. Finally, the speed of the video recording may also change the perception of the raters during the gait cycle analysis (Spitz et al., 2017). Thus, these results suggest that experienced and inexperienced raters can get reliable results if appropriate training is provided. We highly recommended the careful study of the article and videos, as previously published by Metz and Whishaw (2002, 2009), and supervised practice before using the foot fault scoring system.

Despite being originally designed for rats, the LRWT can be used in mice with some adjustments to the apparatus, namely, (a) the diameter of the rungs should be reduced to allow a proper grip and paw placement; and (b) the minimal and maximal between-rung interval should be changed, as previously described (Farr et al., 2006; Wearick-Silva et al., 2018). Our findings show that these adaptations are valid to obtain reliable results in C57BL/6 mice and may be valid for other mice strains.

It is important to mention that an adaptation of a ladder beam walking task in mice following spinal cord injury was previously tested in terms of the test reliability (Cummings et al., 2007). However, the authors did not use the foot fault score system (Metz and Whishaw, 2002, 2009). Thus, we cannot directly compare their obtained findings with this study.

This study has some limitations. First, only two rodent strains were assessed. Anyway, the current findings provide evidence of the accuracy and reliability of the foot fault score in both Wistar rats and C57BL/6 mice. Second, we did not compare specific injury models. Despite this, all individual scores (0–6) in the foot fault score were found in the study videos, which minimize this concern. In this study, a third rater analyzed five of the videos, randomly selected, and all the obtained results were within the limits of the 95% confidence intervals (obtained by raters I and II). Thus, to reach the study goal, we concluded that including more raters would be unnecessary. Nevertheless, the diversity of the rater backgrounds, e.g., researchers from different countries or areas of expertise, might have some influence on the foot fault score reliability.

We concluded that the foot fault score of the LRWT is a reliable and useful tool to study skilled walking in rodents. Damage to the nervous system frequently impairs walking adaptability and performance. When assessing the impact of such damage, the foot fault score has been proven useful in detecting skilled walking deficits in rodent models. Our findings show that experienced and inexperienced raters can obtain reliable results when previous supervised training is provided. These findings are of importance for researchers working in the field of translational neuroscience and motor control and impact on the comparability of results obtained worldwide using the foot fault score in the LRWT.

## Data Availability Statement

The raw data supporting the conclusions of this article will be made available by the authors, without undue reservation.

## Ethics Statement

The animal study was reviewed and approved by the CEUA – Pontifícia Universidade Católica do Rio Grande do Sul.

## Author Contributions

LM, AS, LX, and RM contributed to the conception and design of the study. LM and AS organized the data and performed the statistical analysis. LM, AS, and RM wrote the manuscript. All authors contributed to the manuscript review, read, and approved the submitted version.

## Conflict of Interest

The authors declare that the research was conducted in the absence of any commercial or financial relationships that could be construed as a potential conflict of interest.

## Publisher’s Note

All claims expressed in this article are solely those of the authors and do not necessarily represent those of their affiliated organizations, or those of the publisher, the editors and the reviewers. Any product that may be evaluated in this article, or claim that may be made by its manufacturer, is not guaranteed or endorsed by the publisher.
